# Cerebral venous disorders: clinical presentation, diagnostic strategy, and contemporary management

**DOI:** 10.3389/fneur.2026.1854875

**Published:** 2026-06-19

**Authors:** Anthony P. Terraciano, Khushal Gupta, David J. Altschul

**Affiliations:** Department of Neurological Surgery, Montefiore Medical Center, Albert Einstein College of Medicine, Bronx, NY, United States

**Keywords:** cavernous sinus thrombosis (CST), cerebral venous thrombosis (CVT), dural arteriovenous fistula (DAVF), endovascular neurology, idiopathic intracranial hypertension (IIH), pulsatile tinnitus, venous sinus stenosis, venous sinus stenting (VSS)

## Abstract

Cerebral venous disorders encompass a heterogeneous group of conditions ranging from acute cerebral venous thrombosis to chronic intracranial venous hypertension and stenotic outflow states. Although historically considered uncommon, increasing recognition, improved imaging techniques, and expanding endovascular therapies have led to significant advances in the diagnosis and management of these conditions. This review provides a contemporary overview of the pathophysiology, clinical presentation, diagnostic evaluation, and treatment strategies for major cerebral venous disorders, with an emphasis on conditions associated with elevated intracranial venous pressure and medically refractory symptoms. We summarize current evidence regarding noninvasive and invasive imaging modalities, including the evolving roles of CT and MR venography, catheter venography, and physiologic pressure measurements. Indications for medical therapy, including anticoagulation and intracranial pressure management, are discussed alongside emerging criteria for patient selection for endovascular intervention. Particular attention is given to venous sinus stenting and other endovascular techniques, including technical considerations, outcomes, and complication profiles. Controversies remain regarding diagnostic thresholds, the clinical significance of venous stenosis, optimal hemodynamic criteria for intervention, and long-term durability of endovascular treatment. We highlight areas of ongoing investigation and identify key knowledge gaps that may inform future research. As diagnostic capabilities and therapeutic options continue to evolve, a multidisciplinary and physiology-guided approach is essential to optimize patient selection and outcomes in cerebral venous disorders.

## Introduction

The cerebral venous system drains blood from the brain through a low-pressure network of cortical and deep veins that converge into the dural venous sinuses and ultimately through the jugular venous system back to the right atrium. Because venous anatomy is variable and flow is highly collateralized, venous pathology can present subtly, evolve subacutely, and can be difficult to distinguish from normal variants on imaging. Cerebral venous disorders comprise a heterogeneous group of conditions involving the dural venous sinuses, cortical and deep cerebral veins, and intracranial venous outflow pathways. Although individually uncommon, these entities collectively represent an important and often underrecognized cause of neurologic morbidity, particularly among younger patients. Major categories include thrombotic, infectious, stenotic, shunting, and structural disorders of the intracranial venous system (1). These entities are increasingly encountered as venous imaging becomes more widely used and awareness of treatable venous etiologies increases. Nonetheless, diagnosis remains challenging: presentations are frequently nonspecific (e.g., headache, visual symptoms, pulsatile tinnitus, encephalopathy, focal deficits), and artifacts or anatomic variants may mimic or mask disease (2, 3).

Pathophysiologically, impaired venous drainage produces two major consequences. Venous hypertension may result in intracranial pressure elevation with papilledema and risk of irreversible vision loss. Alternatively, focal venous congestion can cause parenchymal injury, including hemorrhagic venous infarction or intracranial hemorrhage. These manifestations may mimic arterial stroke but require a distinct diagnostic and therapeutic approach (1, 4). Even with contemporary therapy, a subset of patients experience persistent symptoms, cognitive sequelae, or reduced quality of life, underscoring the need for practical frameworks that promote early clinical suspicion, appropriate venous imaging, and standardized interpretation (1, 3).

This review offers a practical overview of major intracranial venous disorders, focusing on diagnosis and management. We cover cerebral venous thrombosis (CVT), cavernous sinus thrombosis (CST), dural arteriovenous fistulas (dAVF), venous sinus stenosis (VSS)/idiopathic intracranial hypertension (IIH), and developmental venous anomalies (DVA), with emphasis on adult presentations (1, 5, 6).

## Methods

A comprehensive literature search was performed in PubMed on February 1, 2026, to identify peer-reviewed studies relevant to cerebral venous disorders, including clinical presentation, diagnostic strategies, and contemporary management. Medical Subject Headings (MeSH) and free-text terms were combined in various permutations, including: “cerebral venous thrombosis,” “cerebral venous sinus thrombosis,” “dural venous sinus,” “cortical vein thrombosis,” “deep cerebral venous thrombosis,” “cavernous sinus thrombosis,” “septic lateral sinus thrombosis,” “dural arteriovenous fistula,” “cortical venous reflux,” “venous hypertension,” “hemorrhagic venous infarction,” “CT venography,” “MR venography,” “digital subtraction angiography,” “venous sinus stenosis,” “transverse sinus stenosis,” “idiopathic intracranial hypertension,” “venous sinus stenting,” and “developmental venous anomaly.” The search was limited to English-language publications. Studies were included if they: (1) addressed intracranial venous disorders involving the dural sinuses, cortical veins, deep venous system, or clinically relevant venous outflow pathways; (2) reported on clinical manifestations, diagnostic performance or diagnostic pathways (including CTV, MRV, MRI, and/or DSA), risk stratification, natural history, or treatment outcomes (medical, surgical, and/or endovascular); (3) involved human subjects (adult and/or pediatric) or authoritative guidelines, consensus statements, and high-quality systematic reviews or meta-analyses; and (4) provided sufficient methodological and clinical detail to inform practical diagnostic and management frameworks. Articles were excluded if they: (1) focused primarily on extracranial jugular venous disease, spinal venous disorders, or non-neurovascular venous conditions outside the scope of intracranial venous pathology; (2) did not provide actionable information on diagnosis or management (e.g., minimal clinical/imaging detail); or (3) were conference abstracts, editorials, or single-patient case reports.

## Venous anatomy and pathophysiologic principles

The cerebral venous system is a valveless, low-pressure network with substantial anatomic variability and the capacity for bidirectional flow. Superficial cortical veins drain primarily into the superior sagittal sinus, whereas deep structures empty through the internal cerebral veins into the vein of Galen and the straight sinus. These channels converge posteriorly at the torcular herophili and ultimately drain through the transverse and sigmoid sinuses into the internal jugular veins (4, 7). The absence of valves facilitates collateral recruitment, contributing to the often subacute and heterogeneous clinical presentation of cerebral venous disorders.

**Figure 1:**
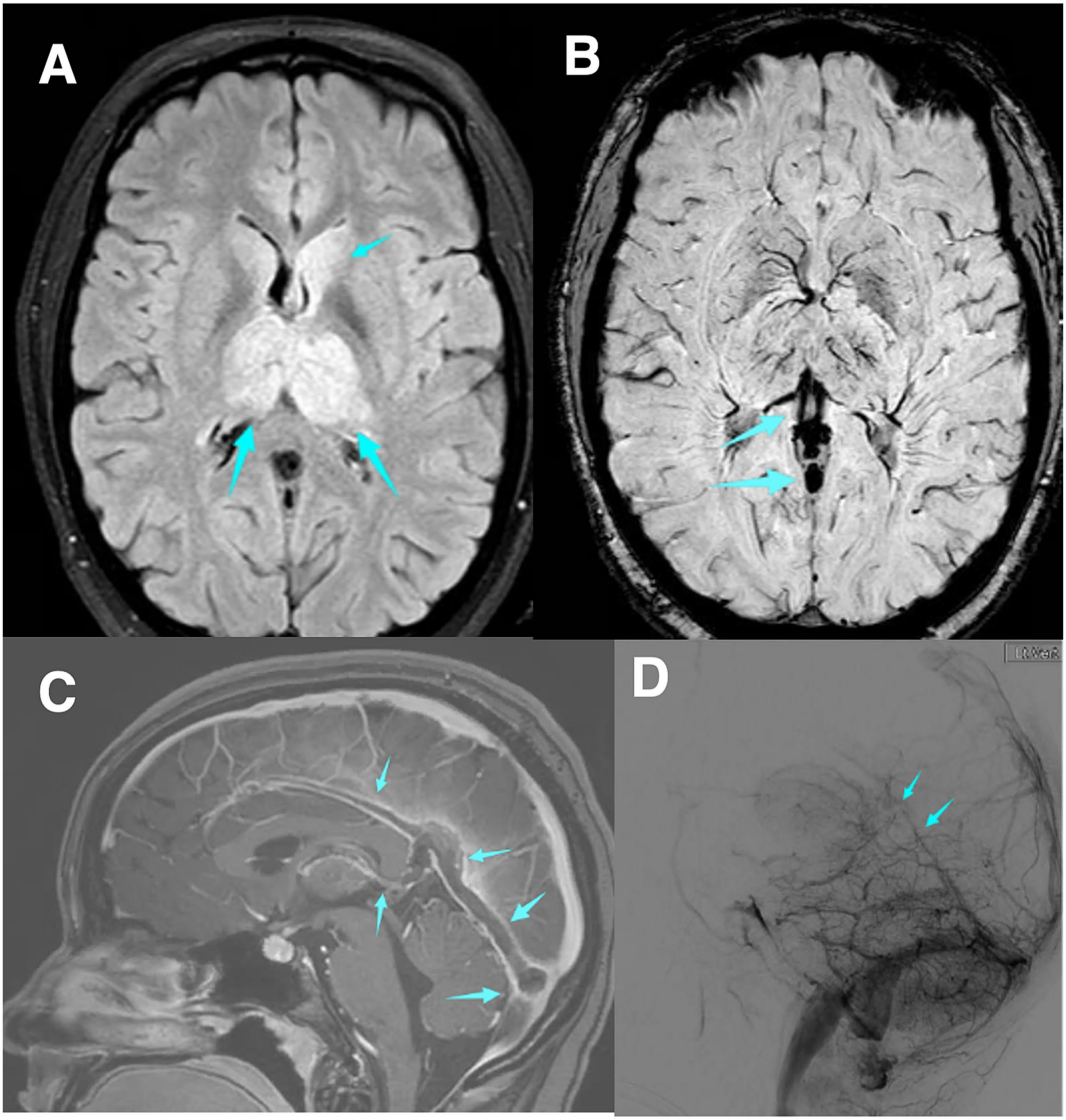
Deep cerebral venous thrombosis with multimodal imaging findings. **(A)** Axial FLAIR demonstrating bilateral thalamic hyperintensity with associated edema extending into the caudate nucleus, as indicated by arrows. **(B)** Susceptibility-weighted imaging showing dilation of the deep venous system consistent with thrombosis. **(C)** Sagittal T1-weighted post-contrast imaging demonstrating absence of enhancement in the vein of Galen, inferior sagittal sinus, and straight sinus. **(D)** Catheter angiography demonstrating impaired deep venous outflow consistent with thrombosis.

**Figure 2:**
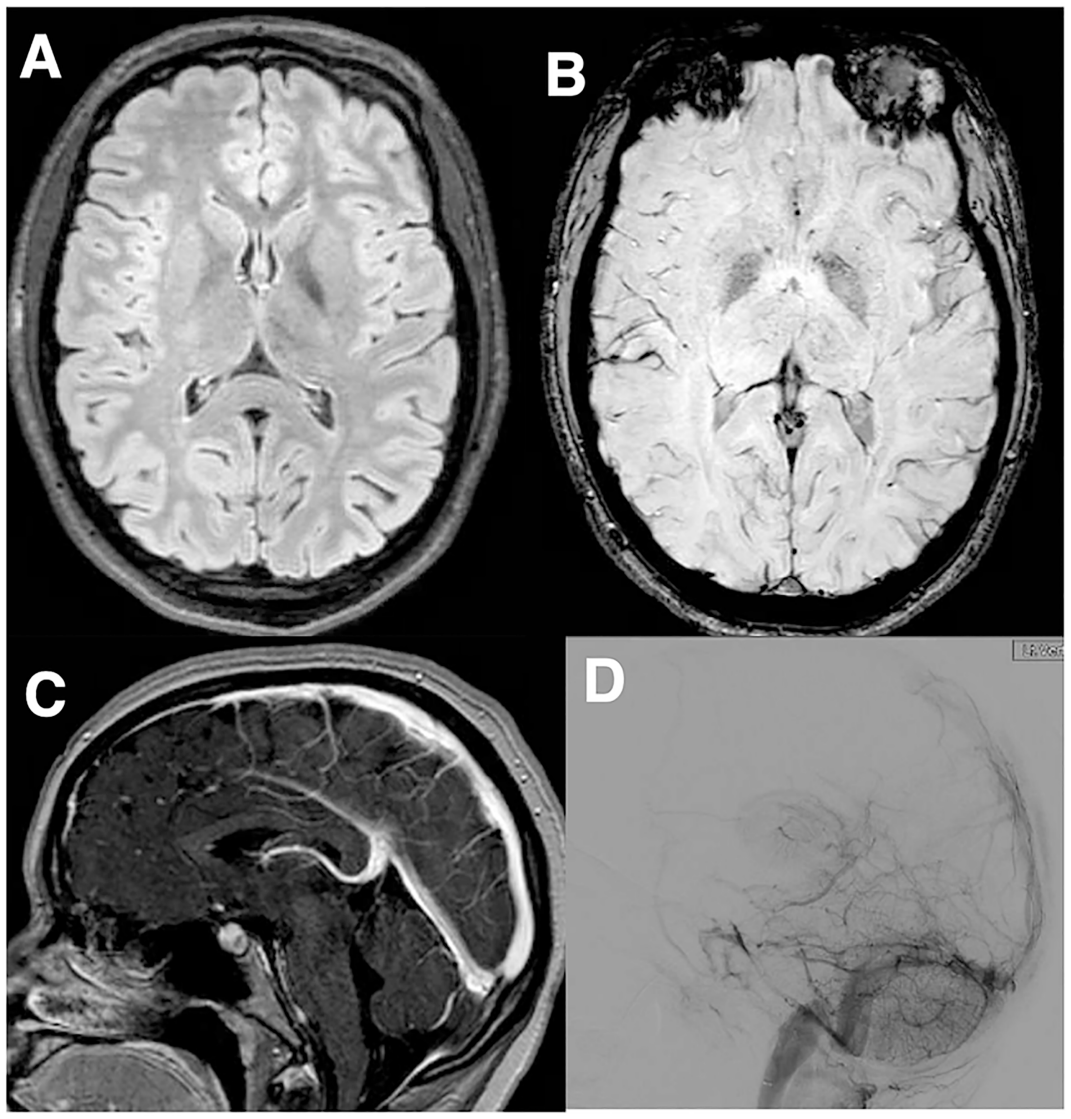
Post-treatment imaging following mechanical thrombectomy and anticoagulation. **(A)** Axial FLAIR showing interval improvement in bithalamic edema. **(B)** Susceptibility-weighted imaging demonstrating interval development of a hemorrhagic venous infarct. **(C)** Sagittal post-contrast imaging demonstrating restored venous opacification. **(D)** Post-treatment angiography demonstrating improved deep venous outflow.

Across disease states, cerebral venous pathology is driven by two interrelated mechanisms: venous hypertension with intracranial pressure elevation and focal parenchymal injury resulting from cortical venous congestion (1, 7). Outflow obstruction from thrombosis or fixed stenosis increases venous pressure, impairs cerebrospinal fluid resorption at the arachnoid granulations, and produces intracranial hypertension without true obstructive hydrocephalus, typically manifesting as progressive headache, papilledema, and visual symptoms. When cortical veins are involved, reduced capillary perfusion and blood–brain barrier disruption lead to combined vasogenic and cytotoxic edema with a high propensity for hemorrhagic venous infarction, a distinguishing feature of venous compared with arterial ischemia (4, 7). Venous congestion also promotes endothelial dysfunction and a local prothrombotic milieu, consistent with the principles of Virchow’s triad (8). The major pathophysiologic mechanisms underlying cerebral venous disorders include thrombotic, shunting, stenotic/outflow, and structural processes. Figures 1, 2 demonstrate representative multimodal imaging findings in deep cerebral venous thrombosis before and after treatment.

### Cerebral venous thrombosis

Cerebral venous thrombosis (CVT) involves thrombosis of the dural sinuses and/or cerebral veins, resulting in impaired venous outflow, venous hypertension, intracranial pressure elevation, and a risk of hemorrhagic venous infarction.

#### Epidemiology and risk factors

Cerebral venous thrombosis accounts for approximately 0.5 to 3% of cases and occurs predominantly in younger patients (1, 2). Reported incidence has increased in recent decades, likely reflecting improved detection with widespread use of CT and MR venography (9). Women are affected more frequently than men, with an approximate 3:1 ratio driven largely by sex-specific risk factors (1, 2).

An identifiable provoking factor or prothrombotic condition is present in most patients. Transient risk factors include pregnancy and the puerperium, oral contraceptive use, trauma, infection, and dehydration, whereas persistent risk factors include inherited or acquired thrombophilias, malignancy, hematologic disorders, and systemic inflammatory or vasculitic conditions (2, 10). Emerging associations include obesity, polycystic ovary syndrome, COVID-19 infection, and vaccine-induced thrombotic thrombocytopenia (11).

#### Clinical presentation

Clinical manifestations of CVT are heterogeneous, reflecting both intracranial hypertension and focal parenchymal injury. Presentations range from isolated headache to rapidly progressive encephalopathy and coma (7). The clinical course is typically subacute, with many patients presenting days after symptom onset, although acute presentations may occur, including thunderclap headache or syndromes mimicking subarachnoid hemorrhage (12).

Headache is the most common symptom, occurring in approximately 90% of patients (12). Seizures are reported in 20–40% of cases, and focal neurologic deficits occur in roughly 20–50% (7). Features of intracranial hypertension include papilledema, transient visual obscurations, and diplopia, most commonly due to abducens nerve palsy (1).

Anatomic extent of thrombosis influences clinical severity and prognosis. Involvement of multiple sinuses and depressed level of consciousness at presentation are consistently associated with worse outcomes, and intracerebral hemorrhage is a marker of severe disease (1, 12). The superior sagittal sinus is most frequently affected, followed by the transverse and sigmoid sinuses (12).

#### Deep cerebral venous thrombosis

Thrombosis involving the internal cerebral veins, vein of Galen, or straight sinus represents a high-risk CVT phenotype associated with increased morbidity and mortality. Deep venous outflow obstruction commonly produces bilateral thalamic edema or infarction, often with hemorrhagic transformation, and may present with rapidly progressive encephalopathy, impaired arousal, or coma (2). MRI typically demonstrates characteristic bilateral thalamic signal abnormalities, sometimes with midbrain extension, which should prompt consideration of deep venous thrombosis in the appropriate clinical setting (3). Management follows general CVT principles with urgent anticoagulation and aggressive neurocritical care (13, 14).

#### Acute management

Therapeutic anticoagulation is the cornerstone of treatment for cerebral venous thrombosis and should be initiated promptly once the diagnosis is established, including in the presence of hemorrhagic venous infarction, as hemorrhage reflects venous hypertension and blood–brain barrier disruption rather than a primary bleeding diathesis (1, 10, 15). Initial therapy typically consists of low-molecular-weight heparin, which is preferred in the absence of contraindications, or unfractionated heparin when rapid reversal may be required, followed by transition to oral anticoagulation. Although vitamin K antagonists have historically been standard therapy, direct oral anticoagulants are increasingly used in appropriately selected patients, with observational data suggesting comparable safety and efficacy (1, 2).

Randomized and prospective data have further informed contemporary management. The RE-SPECT CVT and SECRET trials demonstrated comparable safety and efficacy of direct oral anticoagulants relative to warfarin in selected patients with CVT, supporting their increasing use in clinical practice (16, 17). In contrast, the TO-ACT trial evaluating endovascular therapy did not show a significant functional benefit over standard anticoagulation, although it was limited by early termination and modest sample size (18). Pediatric data from the EINSTEIN Jr. program similarly support the safety of DOACs in children with venous thromboembolism, including CVT, although extrapolation to adult practice should be made cautiously (19).

Management of elevated intracranial pressure is critical and may include head elevation, osmotic therapy, cerebrospinal fluid diversion, or decompressive hemicraniectomy in cases of malignant edema or impending herniation (1, 7). Antiepileptic therapy is recommended for patients presenting with seizures or supratentorial lesions.

Close clinical monitoring is essential. Serial neurologic examinations and repeat imaging are used to detect clinical deterioration, infarct progression, or hemorrhagic expansion. Endovascular therapy, including mechanical thrombectomy or catheter-directed thrombolysis, may be considered in carefully selected patients with clinical deterioration despite adequate anticoagulation, particularly in those with symptomatic deep venous system thrombosis, severely depressed mental status or coma, thrombus propagation, or worsening intracranial hemorrhage. Current guidance from the American Heart Association supports consideration of endovascular intervention as a rescue therapy in patients who fail standard anticoagulation or develop life-threatening mass effect. However, the randomized TO-ACT trial did not demonstrate improved functional outcomes with endovascular therapy compared with anticoagulation alone, and was terminated early for futility, underscoring the need for careful patient selection and the limited strength of current evidence (1, 14).

#### Long-term management and secondary prevention

The duration of anticoagulation after CVT is individualized based on provoking factors and risk of recurrence. A limited course (typically 3–6 months) is appropriate for provoked events, whereas extended or indefinite therapy may be considered for unprovoked thrombosis, recurrent events, or high-risk thrombophilia (1, 2). Evaluation for underlying prothrombotic conditions is reasonable in selected patients after the acute phase.

Secondary prevention should address modifiable risk factors, including discontinuation of estrogen-containing therapies, treatment of infection or dehydration, weight reduction in patients with obesity or IIH, and counseling regarding future pregnancy and thrombosis risk. Follow-up venous imaging is commonly performed at 3–6 months to assess recanalization and exclude underlying structural pathology, although the degree of recanalization correlates imperfectly with clinical recovery.

#### Outcomes

Overall prognosis after CVT is favorable, with approximately 70–80% of patients achieving complete or near-complete functional recovery. Contemporary mortality ranges from 3 to 10%, although death or long-term dependence occurs in roughly 10–15% of patients in modern series (2). Predictors of poor outcome include older age, decreased level of consciousness or coma at presentation, deep venous system involvement, intracerebral hemorrhage, posterior fossa lesions, active malignancy, and extensive sinus thrombosis (1).

Despite generally good functional recovery, persistent symptoms are common, including chronic headache, fatigue, cognitive impairment, and reduced quality of life. (1) Venous recanalization occurs in most patients (approximately 70–90%), but the degree of recanalization correlates imperfectly with clinical outcome, reflecting the importance of collateral circulation and the extent of parenchymal injury. (2) Recurrent CVT or other venous thromboembolic events occur in a minority of patients, with recurrence rates of approximately 2–7% over several years and higher risk among those with persistent thrombophilia or malignancy (15).

### Cavernous sinus thrombosis

Cavernous sinus thrombosis (CST) most commonly arises as a septic thrombophlebitis from contiguous infection, but noninfectious etiologies including trauma, surgery, hypercoagulable states, and malignancy have also been described. Although uncommon, CST is a medical emergency associated with high morbidity due to cranial neuropathies, vision loss, and intracranial complications, requiring prompt antimicrobial therapy and source control.

#### Epidemiology and risk factors

Cavernous sinus thrombosis is a rare but life-threatening septic thrombophlebitis. Although mortality has declined substantially in the antibiotic era, contemporary series report case-fatality rates of approximately 4 to 8%, and neurological morbidity remains common, affecting 25 to 67% of survivors (6, 20, 21).

Most cases result from contiguous spread of infection from adjacent structures, particularly the paranasal sinuses, orbit, or facial soft tissues. Sphenoid and ethmoid sinusitis are the most frequently implicated sources and typically precede CST by several days (6, 22, 23). Less common etiologies include odontogenic infections, maxillofacial trauma or recent procedures, otomastoiditis, and, rarely, systemic hypercoagulable states or medication-associated thrombosis (6, 24). Diabetes mellitus is a common comorbidity and is present in a substantial proportion of culture-confirmed cases (25).

The microbiology of CST is dominated by *Staphylococcus aureus*, followed by streptococcal species, oral anaerobes, and gram-negative organisms. Fungal pathogens, particularly *Aspergillus*, should be considered in immunocompromised patients (5, 6, 25). These pathogen patterns support early empiric broad-spectrum intravenous antimicrobial therapy pending culture results.

#### Clinical presentation

Cavernous sinus thrombosis typically presents with the triad of fever, headache, and orbital findings (6). Headache and fever are common early symptoms, followed by progressive orbital manifestations including chemosis, periorbital edema, ptosis, proptosis, ophthalmoplegia, and visual disturbance (6, 20).

Cranial neuropathies reflect involvement of structures traversing the cavernous sinus. Cranial nerve VI palsy often occurs early due to its medial course within the sinus, followed by involvement of cranial nerves III and IV and sensory deficits in the ophthalmic (V1) and maxillary (V2) distributions (4). Because the cavernous sinuses communicate through intercavernous channels, bilateral orbital involvement or progression of symptoms to the contralateral eye is highly suggestive of cavernous sinus pathology and should heighten clinical suspicion (22).

Meningismus, altered mental status, or focal neurologic deficits may occur with intracranial extension, septic embolization, or adjacent meningeal inflammation (5). Early recognition is critical, as delays in diagnosis and treatment are consistently associated with worse neurological and visual outcomes (25).

#### Management

Management of cavernous sinus thrombosis focuses on three parallel priorities: prompt antimicrobial therapy, control of the primary infectious source, and prevention of thrombus progression. Broad-spectrum intravenous antibiotics should be initiated immediately and tailored once microbiologic data are available (5, 6). Early source control, including drainage of paranasal sinus, orbital, or facial infection when indicated, is critical to limit ongoing septic propagation (22).

Adjunctive anticoagulation is commonly used to reduce thrombus extension and promote recanalization, although high-quality evidence for improved mortality or functional outcomes remains limited and practice varies across centers (20, 24). Given the risk of rapid neurological deterioration and intracranial complications, close clinical monitoring and multidisciplinary management are essential (6).

#### Outcomes

Outcomes after cavernous sinus thrombosis have improved substantially in the antibiotic era but remain clinically significant. Contemporary series report mortality rates of approximately 4–8%, with persistent neurological or ophthalmologic deficits occurring in 25 to 67% of survivors (6, 20, 21). Common long-term sequelae 1include cranial neuropathies, persistent ophthalmoplegia, visual impairment, and chronic headache (25).

Delayed diagnosis and treatment are consistently associated with worse outcomes, including higher rates of vision loss and intracranial complications (25). Fungal infections, immunocompromised status, and altered mental status at presentation are also associated with more severe disease and increased morbidity and mortality (5, 21).

### Septic lateral sinus thrombosis

Septic lateral sinus thrombosis (SLST) is a form of septic cerebral venous sinus thrombosis involving the sigmoid or transverse sinus that most commonly arises as a complication of acute otitis media or mastoiditis. Although uncommon in the modern antibiotic era, SLST remains an important cause of intracranial morbidity and requires prompt recognition and treatment to prevent intracranial complications (26–29).

#### Epidemiology and risk factors

The incidence of SLST has declined substantially with widespread antibiotic use but continues to occur in patients with complicated otomastoiditis, particularly children and young adults and those with chronic suppurative otitis media (27, 29). Infection typically spreads from the mastoid air cells to the adjacent sigmoid sinus through bony erosion, thrombophlebitis, or emissary venous channels (26–28).

Risk factors include delayed or inadequate treatment of acute otitis media or mastoiditis, chronic ear disease, immunocompromised states, and other conditions associated with impaired host defense (26–29). As with other forms of cerebral venous thrombosis, additional prothrombotic factors may coexist in some patients and should be considered during evaluation (1, 30).

#### Clinical presentation

SLST typically develops in the setting of acute or chronic otomastoiditis and should be suspected in patients with persistent or worsening systemic or neurologic symptoms despite appropriate antimicrobial therapy (26–29). Common presenting features include fever, headache, otalgia or otorrhea, and postauricular pain or tenderness (26–28).

Persistent fever despite treatment is a common clinical clue, and intermittent spiking temperatures (“picket-fence” fever) may be observed but are not consistently present (26–28). Symptoms related to intracranial hypertension or venous congestion can occur, including nausea, vomiting, papilledema, or diplopia (26–28, 31). Neck pain, retroauricular tenderness, or edema over the mastoid region may reflect local disease extension (26–28).

Systemic septic complications such as bacteremia, septic pulmonary emboli, or metastatic infection may occur in advanced cases (26–28). Intracranial complications, including epidural or subdural collections, cerebellar or temporal lobe abscess, and meningitis, may also be present (26–28, 31).

Clinical findings may be subtle, particularly in children, where persistent fever, irritability, vomiting, or new or worsening headache may be the only early indicators (26–28, 32). Because symptoms can be nonspecific, a low threshold for venous imaging is warranted in patients with otomastoiditis who fail to improve or who develop new neurologic or systemic signs (32).

#### Management

Management of SLST focuses on prompt source control and systemic antimicrobial therapy. Broad-spectrum intravenous antibiotics targeting common otogenic pathogens are initiated empirically and tailored based on culture results (26–29, 31). Surgical management with cortical mastoidectomy, often combined with myringotomy or tympanostomy tube placement, is recommended to eradicate the primary infection and prevent further intracranial spread (26–29, 31).

Direct surgical exploration or thrombectomy of the affected sinus is rarely required and is generally reserved for patients with persistent sepsis, large infected thrombus with abscess formation, or clinical deterioration despite appropriate medical and otologic management (27, 29).

The role of systemic anticoagulation remains controversial. Some series report favorable outcomes with antibiotics and mastoidectomy alone, whereas others suggest anticoagulation may help prevent thrombus propagation and facilitate venous recanalization (26–28). Although high-quality evidence specific to septic disease is limited, many centers consider anticoagulation in selected patients after surgical source control, particularly in cases of thrombus extension, intracranial complications, or additional prothrombotic risk factors (1, 17, 28, 31). Management decisions should be individualized based on bleeding risk, surgical considerations, and overall clinical status.

#### Outcomes

With modern antibiotics and timely surgical management, outcomes in septic lateral sinus thrombosis are generally favorable compared with the preantibiotic era. Contemporary series report low mortality, typically below 5%, with most patients achieving good neurological recovery when diagnosis and treatment are prompt (26–29).

Complications remain important and include intracranial abscess, meningitis, venous infarction, hydrocephalus, and septic emboli, particularly to the lungs (26–29, 33). Persistent symptoms such as headache, papilledema, or signs of intracranial hypertension may occur, and a subset of patients develop chronic venous occlusion despite clinical improvement (26–28).

Delayed diagnosis, extensive sinus involvement, intracranial extension of infection, and underlying immunocompromised states are associated with worse outcomes (26–29). Early recognition and aggressive source control remain the most important determinants of prognosis.

### Dural arteriovenous fistulas

dAVFs are acquired vascular shunts between meningeal arteries and dural venous sinuses, meningeal veins, or cortical veins, accounting for approximately 10–15% of intracranial vascular malformations (34, 35). dAVFs are most commonly diagnosed in middle-aged to older adults and may arise in association with prior sinus thrombosis, trauma, surgery, or other causes of dural sinus hypertension. The natural history of dAVFs is determined primarily by their venous drainage pattern. Lesions that drain only into a dural sinus without cortical venous reflux (Borden I/Cognard I–IIa) generally have a benign clinical course, whereas the presence of cortical venous reflux markedly increases the risk of intracranial hemorrhage and non-hemorrhagic neurologic deficits (34, 36).

Increasingly, dAVFs are recognized as dynamic venous disorders in which venous hypertension, sinus thrombosis, and progressive alterations in venous outflow contribute to lesion evolution and clinical deterioration. Advanced venous congestion may produce a pseudophlebitic pattern, characterized by engorged serpiginous medullary veins on imaging, which reflects severe venous hypertension and is associated with aggressive clinical behavior and worse outcomes (36, 37).

#### Clinical presentation

Clinical manifestations depend largely on the location of the fistula and the severity of venous hypertension. Low-grade lesions commonly present with pulsatile tinnitus, cranial bruit, headache, or orbital symptoms related to venous congestion (34, 35). In contrast, high-grade fistulas with cortical venous reflux may present with intracerebral hemorrhage, seizures, progressive focal neurologic deficits, or cognitive decline due to chronic venous ischemia (34, 35).

Among angiographically aggressive lesions, exclusive leptomeningeal venous drainage and the presence of a pseudophlebitic pattern are associated with higher rates of non-hemorrhagic neurologic deficits and progressive encephalopathy (35, 36). Emerging data also suggest that systemic inflammatory markers, including neutrophil-to-lymphocyte ratio and related indices, may correlate with disease severity and functional outcomes, supporting a role for host inflammatory state in shaping the clinical phenotype (38, 39).

#### Diagnosis

Digital subtraction angiography (DSA) remains the gold standard for diagnosis and classification, allowing detailed characterization of arterial supply, venous drainage patterns, and the presence of cortical venous reflux using Borden or Cognard grading systems (34, 40).

Magnetic resonance imaging plays an important complementary role. Susceptibility-weighted imaging (SWI) can detect cortical venous reflux, venous ectasia, and the pseudophlebitic pattern, and may demonstrate parenchymal signal abnormalities related to chronic venous congestion (41, 42). Recent studies suggest that SWI can differentiate aggressive from benign fistulas with high diagnostic accuracy (42). Emerging machine learning approaches applied to susceptibility-weighted sequences have shown promise for automated detection and triage, although these techniques remain investigational (43).

Large multicenter data from the CONDOR registry (44) have further clarified the natural history of dAVFs, demonstrating that cortical venous reflux is the primary determinant of hemorrhage risk and clinical aggressiveness, reinforcing its central role in risk stratification and treatment decision-making.

#### Management

Management is risk-stratified according to venous drainage pattern and clinical presentation. Lesions with cortical venous reflux or symptomatic venous hypertension carry a substantial risk of hemorrhage and neurologic deterioration and require prompt definitive treatment (34, 35). Endovascular therapy is the primary treatment modality and may be performed via transarterial or transvenous approaches depending on fistula anatomy. Transarterial embolization is often favored for lesions with accessible arterial feeders and may achieve cure using liquid embolic agents such as Onyx or n-BCA. Transvenous embolization is typically employed when arterial access is limited or in cases with direct sinus involvement, allowing for coil or liquid embolic occlusion of the venous recipient. Increasingly, a combined or staged approach is used for complex lesions to maximize obliteration rates while minimizing risk. Microsurgical disconnection or stereotactic radiosurgery may be considered for lesions not amenable to endovascular cure or for residual disease (35, 43).

Asymptomatic low-grade fistulas without cortical venous reflux may be managed conservatively with clinical and imaging surveillance. Increasingly, features such as severe venous congestion or elevated inflammatory indices are being explored as potential modifiers of treatment timing and post-treatment monitoring, although their role in routine decision-making remains investigational (38, 40, 41).

#### Outcomes

**Figure 3:**
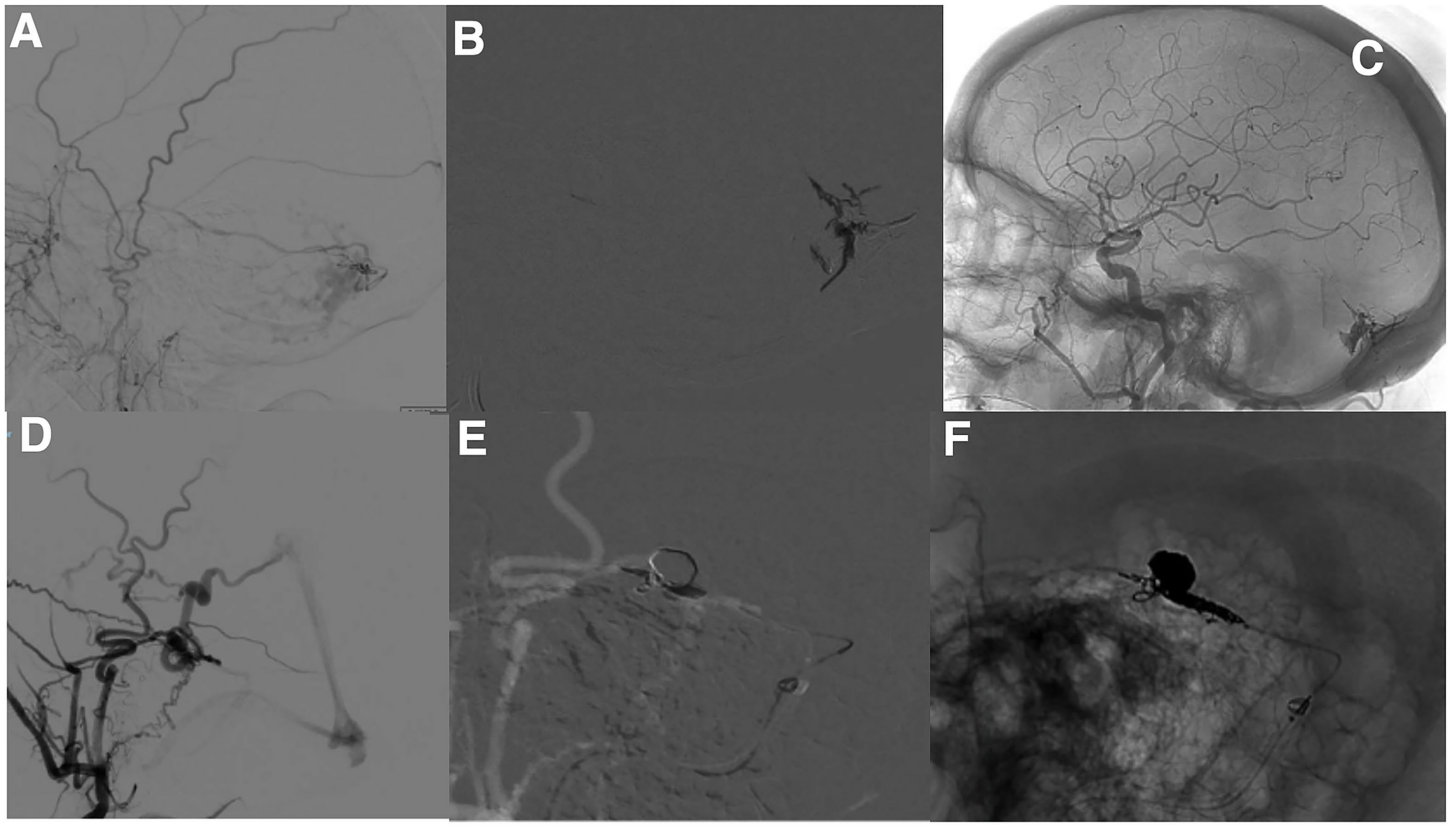
Endovascular treatment approaches for dural arteriovenous fistulas. **(A–C)** Transarterial embolization of a dural arteriovenous fistula with venous ectasia, including diagnostic, selective, and post-embolization angiographic views. **(D–F)** Transvenous embolization of a dural arteriovenous fistula, including catheterization, embolization, and final angiographic result.

Complete angiographic obliteration is associated with symptom resolution and elimination of hemorrhage risk. Endovascular cure rates range from approximately 60 to 90%, depending on lesion complexity and venous anatomy. Recurrence after complete obliteration is uncommon (<5%), but incomplete treatment may worsen venous hemodynamics and should be avoided. Stereotactic radiosurgery achieves obliteration in approximately 60–80% of cases over 2–3 years but carries a latency period during which the annual hemorrhage risk persists at approximately 2–3% (Figure 3).

### Venous sinus stenosis and idiopathic intracranial hypertension (IIH)

Idiopathic intracranial hypertension is characterized by elevated intracranial pressure in the absence of an intracranial mass lesion or abnormal cerebrospinal fluid composition. The disorder classically affects obese women of childbearing age and most commonly presents with headache and papilledema (45, 46).

Although historically considered idiopathic, modern imaging demonstrates VSS in the majority of patients, with reported prevalence of approximately 90–93% (47, 48). Current pathophysiologic models describe IIH as a venous outflow disorder in which elevated intracranial pressure compresses the compliant transverse sinuses, worsening venous hypertension and further impairing cerebrospinal fluid absorption in a self-reinforcing “Starling-like resistor” mechanism (48). Obesity, hormonal influences, and altered CSF or glymphatic dynamics likely contribute to initiation of this cycle (45, 48).

These observations have shifted the conceptual framework of IIH from an isolated intracranial pressure disorder toward a disorder of venous outflow within the broader spectrum of cerebral venous disease (49, 50).

#### Clinical presentation

IIH typically presents with daily or near-daily headache, transient visual obscurations, pulsatile tinnitus, and papilledema (45, 46). Visual symptoms range from transient dimming to progressive visual field loss and represent the major source of long-term morbidity.

Symptom severity correlates with the degree of venous outflow impairment in some series, with more severe or bilateral VSS associated with greater papilledema and visual dysfunction (47). Structured outcome measures, including the proposed Cerebral Venous Disorders Severity Scale (CVDSS), have been developed to quantify symptom burden and treatment response across venous disorders (50, 51).

#### Diagnosis

Diagnosis is based on modified Dandy (Friedman) criteria, including symptoms and signs of intracranial hypertension, elevated lumbar puncture opening pressure with normal CSF composition, and the absence of an alternative structural cause on neuroimaging (46, 52).

MRI may demonstrate supportive findings such as empty sella, globe flattening, or optic nerve sheath distention, while MR venography frequently reveals unilateral or bilateral VSS (47, 48). Intrinsic (arachnoid granulation–related) and extrinsic (pressure-related) stenoses may be distinguished, as they may respond differently to intervention (48).

Catheter venography with manometry remains the reference standard for identifying a physiologically significant trans-stenotic pressure gradient when venous sinus stenting is being considered. Emerging techniques such as 4D-flow MRI allow noninvasive assessment of venous hemodynamics and may improve patient selection and post-treatment evaluation (45, 53).

Initial evaluation of suspected IIH includes exclusion of secondary causes of intracranial hypertension through MRI with venography, followed by lumbar puncture to confirm elevated opening pressure with normal cerebrospinal fluid composition (54, 55). In patients with venous sinus stenosis on noninvasive imaging, catheter venography with manometry is essential to assess for a physiologically significant trans-stenotic pressure gradient, which guides candidacy for venous sinus stenting (52, 56). Pressure measurements are typically obtained across the stenosis, with gradients of 8–10 mm Hg commonly used as a threshold for intervention, although this remains an area of ongoing investigation (57, 58).

#### Management

Initial treatment focuses on weight reduction and medical therapy with carbonic anhydrase inhibitors. The Idiopathic Intracranial Hypertension Treatment Trial demonstrated that acetazolamide combined with a weight-reduction program produces modest but significant improvements in visual function and papilledema in patients with mild visual loss (45, 59).

When vision is threatened or symptoms are refractory, surgical options include cerebrospinal fluid diversion or optic nerve sheath fenestration (52).

Venous sinus stenting has emerged as an important therapy for selected patients with VSS and a demonstrable pressure gradient. Observational series report improvement in papilledema, visual function, pulsatile tinnitus, and headache, along with reductions in intracranial pressure (45, 53, 60). Dedicated venous stent systems are currently under investigation, including in the RIVER trial (61). Despite favorable short-term outcomes, restenosis, symptom recurrence, and ongoing need for medical therapy may occur in a subset of patients (62, 63).

Weight loss remains the cornerstone of therapy in patients with underlying obesity. Patients with primary venous sinus stenosis in the absence of obesity may derive greater benefit from endovascular intervention, suggesting heterogeneity in disease mechanisms.

#### Outcomes

Visual preservation is the primary treatment goal, as approximately 5–10% of patients develop severe permanent vision loss despite therapy (45, 46). Weight loss produces sustained improvement in many patients who achieve and maintain target reduction, and medical therapy alone controls symptoms in approximately 60–70% (45).

Cerebrospinal fluid shunting provides rapid pressure control but is associated with high revision rates, with 30–50% requiring reoperation within 5 years (52).

Venous sinus stenting studies report symptomatic improvement in approximately 70–90% of appropriately selected patients, although available data are limited by selection bias and relatively short follow-up. Restenosis or recurrent symptoms occur in approximately 10–20% of cases (53, 60–64).

### Pulsatile tinnitus and venous etiologies

Pulsatile tinnitus (PT) is an increasingly recognized manifestation of intracranial venous pathology and represents the most common identifiable cause of vascular tinnitus. Venous etiologies include venous sinus stenosis, dural arteriovenous fistulas (dAVFs), and sigmoid sinus wall abnormalities, including diverticulum and dehiscence. Unlike non-pulsatile tinnitus, venous PT is often treatable, and in many cases curable, making accurate diagnosis and targeted intervention critical (65, 66).

#### Clinical presentation

Patients typically describe a unilateral or bilateral pulse-synchronous “whooshing” sound that may fluctuate with head position, Valsalva maneuver, or ipsilateral jugular compression, the latter representing a key clinical clue to a venous origin. PT may occur in isolation or in association with symptoms of intracranial hypertension, particularly in patients with coexisting idiopathic intracranial hypertension (IIH), in whom tinnitus is reported in up to 60% of cases (65, 67). Physical examination should include auscultation for objective bruits and assessment for papilledema or other signs of elevated intracranial pressure.

#### Diagnosis

A stepwise diagnostic approach progressing from noninvasive to invasive imaging is recommended. MRI with MR venography serves as the preferred initial modality, allowing evaluation for venous sinus stenosis, dAVF, and intracranial hypertension without radiation exposure (65, 68). CT venography and high-resolution temporal bone CT provide complementary assessment of bony and venous anatomy, particularly for detecting sigmoid sinus wall abnormalities and quantifying stenosis severity (69, 70). Digital subtraction angiography remains the gold standard when dAVF is suspected or when noninvasive imaging is inconclusive, enabling definitive characterization of venous drainage patterns and facilitating therapeutic planning (68, 71, 72). In selected patients, catheter venography with manometry is performed to assess trans-stenotic pressure gradients, with thresholds of 8–10 mm Hg commonly used to define hemodynamic significance (73, 74).

#### Management

Management is directed at the underlying venous pathology and requires careful correlation between clinical presentation, imaging findings, and hemodynamic assessment. In patients with venous sinus stenosis and a demonstrable pressure gradient, venous sinus stenting has emerged as an effective minimally invasive treatment, with pooled data demonstrating symptom improvement in over 90% of cases and complete resolution in approximately 85–90% (75). Endovascular embolization remains the treatment of choice for dAVF-associated PT and can provide definitive symptom relief when complete fistula occlusion is achieved (76, 77). Sigmoid sinus wall abnormalities may be managed with surgical resurfacing or, in selected cases, endovascular approaches, particularly when coexisting venous sinus stenosis is present (70, 72). Optimal outcomes depend on appropriate patient selection, including concordance between symptom laterality and imaging findings and failure of conservative management.

#### Outcomes

Endovascular treatment of venous PT is associated with high rates of durable symptom resolution and low complication rates. Venous sinus stenting demonstrates favorable safety profiles, with near-zero permanent morbidity and low recurrence rates, although recurrence is more common in patients with underlying IIH (73, 75). Similarly, endovascular treatment of dAVFs achieves high rates of symptom resolution when complete occlusion is obtained (77). Despite these favorable outcomes, persistent or recurrent symptoms may occur in patients with multifactorial venous pathology or incomplete hemodynamic correction, underscoring the importance of comprehensive evaluation and longitudinal follow-up.

### Structural variants and secondary venous compression

Some venous abnormalities represent normal anatomic variants or secondary effects of adjacent pathology rather than primary venous disease. Distinguishing primary venous pathology from normal variants or secondary changes is an important diagnostic consideration, as misclassification may lead to unnecessary or inappropriate intervention. Accurate interpretation requires integration of clinical context, imaging findings, and, when relevant, physiologic assessment (78).

#### Developmental venous anomalies

Developmental venous anomalies (DVAs) are common congenital variants of cerebral venous drainage and are most often incidental findings. These structures represent functional venous outflow pathways, and interruption of the draining vein can result in venous infarction or hemorrhage. Accordingly, DVAs are generally managed conservatively and are not treated in isolation (78).

When a DVA is associated with a symptomatic cavernous malformation, management is typically directed toward the cavernoma while preserving the anomalous venous drainage whenever possible, given the critical role of the DVA in regional venous outflow (79).

#### Secondary venous sinus compression

Venous sinus narrowing may also occur as a secondary phenomenon due to extrinsic compression from adjacent pathology, including tumors (most commonly meningiomas), postoperative changes, trauma, or inflammatory processes. In these settings, the venous abnormality reflects the underlying condition rather than primary venous disease (80, 81).

Management generally focuses on treatment of the causative pathology, which may include surgical resection, radiotherapy, or systemic oncologic therapy. Anticoagulation may be considered in selected patients to reduce the risk of superimposed thrombosis. In cases of persistent symptomatic venous outflow obstruction with a demonstrated physiologically significant gradient, venous sinus stenting has been reported as a potential adjunctive treatment option (82, 83).

## Discussion: controversies and knowledge gaps

### Cerebral venous thrombosis

Despite advances in diagnosis and treatment, several areas of uncertainty remain across cerebral venous disorders. The role of direct oral anticoagulants (DOACs) in cerebral venous thrombosis (CVT) continues to evolve. Recent randomized and observational data suggest comparable efficacy to vitamin K antagonists with lower rates of major hemorrhage, and current guidelines consider DOACs a reasonable option after initial parenteral anticoagulation. However, optimal patient selection, timing of initiation, and use in populations excluded from existing studies, including pregnancy, malignancy, and antiphospholipid syndrome, remain uncertain (84).

Management of hemorrhagic venous infarction remains another area of clinical tension. Although anticoagulation is recommended even in the presence of intracerebral hemorrhage, consistent with guideline-supported evidence demonstrating overall safety and benefit, uncertainty persists in patients with large parenchymal hematomas or impending mass effect, where the balance between thrombus control and hemorrhagic expansion is less clearly defined (1).

Seizure prevention strategies in CVT also lack high-quality evidence. Current recommendations advise against routine prophylactic antiepileptic therapy in patients without seizures, yet risk stratification based on lesion location, hemorrhage, and cortical involvement remains derived largely from observational data rather than randomized studies (10, 85).

### Idiopathic intracranial hypertension and venous sinus stenosis

In IIH, venous sinus stenting has emerged as an effective treatment for selected patients with venous outflow obstruction, but important questions remain regarding optimal candidacy criteria and long-term durability. Most centers use a trans-stenotic pressure gradient threshold of approximately 8–10 mm Hg to guide intervention, although recent data suggest that some patients with lower gradients may also benefit, highlighting limitations of current physiologic thresholds (49, 86). While short-term outcomes are favorable, restenosis or adjacent stenosis occurs in a meaningful minority of patients, and long-term comparative data with cerebrospinal fluid diversion or medical therapy are limited (60).

More broadly, improved physiologic biomarkers of clinically significant venous hypertension are needed. Anatomic stenosis on imaging correlates imperfectly with symptoms and treatment response, and emerging tools such as advanced venous flow imaging and refined manometric assessment may improve patient selection for intervention. Finally, heterogeneity in outcome definitions, including recanalization, symptom resolution, and functional recovery, limits cross-study comparison and highlights the need for standardized reporting frameworks across cerebral venous disorders. The role of venous sinus stenting in patients with isolated pulsatile tinnitus without classic IIH features remains an area of ongoing investigation.

### Dural arteriovenous fistulas

In dAVFs, uncertainties remain regarding optimal timing of intervention in minimally symptomatic patients without cortical venous reflux, as well as the role of emerging biomarkers and advanced imaging features in predicting lesion progression.

## Conclusion

Cerebral venous disorders represent a heterogeneous but increasingly recognized group of conditions unified by the consequences of impaired venous outflow, venous hypertension, and secondary parenchymal or visual injury, as well as disabling symptoms such as pulsatile tinnitus. Although individual entities such as cerebral venous thrombosis, dAVFs, and VSS differ in etiology and management, their clinical presentations often overlap and are frequently subacute or nonspecific, contributing to delayed diagnosis. Early recognition, appropriate venous imaging, and an understanding of disease-specific risk features are essential to prevent irreversible neurological or visual morbidity. Advances in neuroimaging, endovascular therapy, and physiologic assessment have expanded treatment options and reframed many of these conditions as treatable disorders of venous hemodynamics rather than isolated structural abnormalities. Despite these advances, important gaps remain in risk stratification, patient selection for intervention, and long-term outcome data. A practical, unified clinical framework, combined with multidisciplinary collaboration among neurology, neurosurgery, neurointerventional, neuroradiology, and neuro-ophthalmology specialists, is essential to optimize diagnosis, guide management, and improve outcomes as the field continues to evolve.

### Key points for clinical practice

Cerebral venous pathology should be considered in patients presenting with headache accompanied by seizures, hemorrhagic infarction that does not conform to arterial territories, papilledema with pulsatile tinnitus, or stroke-like symptoms in younger individuals with risk factors such as pregnancy, thrombophilia, or malignancy. Both CT venography and MR venography are effective diagnostic modalities, although each has limitations, and repeat or complementary imaging should be considered when clinical suspicion remains high despite negative initial studies.

Prompt therapeutic anticoagulation is the cornerstone of treatment for CVT and should be initiated in most patients, including those with hemorrhagic venous infarction, as the benefits generally outweigh the risk of hemorrhagic progression. Deep venous system involvement is associated with worse outcomes and warrants close monitoring, early neurocritical care, and consideration of escalation strategies in deteriorating patients.

Disease-specific risk stratification is essential. dAVFs require angiographic characterization, as the presence of cortical venous reflux confers a high risk of hemorrhage and mandates definitive treatment. In IIH, management should prioritize preservation of vision through weight reduction and medical therapy, with surgical intervention or venous sinus stenting considered in medically refractory cases after careful physiologic evaluation.

Developmental venous anomalies represent normal variants of venous drainage and should not be treated in isolation, as interruption of the draining vein may result in venous infarction. The duration of anticoagulation after CVT should be individualized based on provoking factors, thrombophilia, and recurrence risk, typically ranging from three to twelve months, with longer therapy for unprovoked or recurrent events.

Importantly, radiographic recanalization correlates imperfectly with clinical recovery, reflecting the importance of collateral development and parenchymal injury rather than venographic appearance alone. Given the complexity and multisystem nature of cerebral venous disorders, multidisciplinary collaboration among neurology, neurosurgery, neurointerventional, neuroradiology, and neuro-ophthalmology specialists is critical to optimize patient outcomes.

Pulsatile tinnitus that is pulse-synchronous and improves with ipsilateral jugular compression should prompt evaluation for venous etiologies, including venous sinus stenosis and dural arteriovenous fistula, as these conditions are often amenable to endovascular treatment.

## References

[1] Saposnik G Bushnell C Coutinho JM Field TS Furie KL Galadanci N . Diagnosis and management of cerebral venous thrombosis: a scientific statement from the American Heart Association. Stroke. (2024) 55:e77–90. doi: 10.1161/STR.0000000000000456, 38284265

[2] Borhani-Haghighi A Hooshmandi E. Cerebral venous thrombosis: a practical review. Postgrad Med J. (2024) 100:68–83. doi: 10.1093/postmj/qgad103, 37978050

[3] Spadaro A Scott KR Koyfman A Long B. Cerebral venous thrombosis: diagnosis and management in the emergency department setting. Am J Emerg Med. (2021) 47:24–9. doi: 10.1016/j.ajem.2021.03.040, 33765589

[4] Ropper AH Klein JP. Cerebral venous thrombosis. N Engl J Med. (2021) 385:59–64. doi: 10.1056/NEJMra2106545 34192432

[5] Caranfa JT Yoon MK. Septic cavernous sinus thrombosis: a review. Surv Ophthalmol. (2021) 66:1021–30. doi: 10.1016/j.survophthal.2021.03.009, 33831391

[6] Long B Field SM Singh M Koyfman A. High risk and low prevalence diseases: cavernous sinus thrombosis. Am J Emerg Med. (2024) 83:47–53. doi: 10.1016/j.ajem.2024.06.024, 38959601

[7] Stam J. Thrombosis of the cerebral veins and sinuses. N Engl J Med. (2005) 352:1791–8. doi: 10.1056/NEJMra042354, 15858188

[8] Zhou C Zhou Y Ma W Liu L Zhang W Li H . Revisiting Virchow's triad: exploring the cellular and molecular alterations in cerebral venous congestion. Cell Biosci. (2024) 14:131. doi: 10.1186/s13578-024-01314-5, 39444013 PMC11515517

[9] Capecchi M Abbattista M Martinelli I. Cerebral venous sinus thrombosis. J Thromb Haemost. (2018) 16:1918–31. doi: 10.1111/jth.14210 29923367

[10] Saposnik G Barinagarrementeria F Brown RD Jr Bushnell CD Cucchiara B Cushman M . Diagnosis and management of cerebral venous thrombosis: a statement for healthcare professionals from the American Heart Association/American Stroke Association. Stroke. (2011) 42:1158–92. doi: 10.1161/STR.0b013e31820a8364, 21293023

[11] Dix C Hunt BJ. The changing face of cerebral venous sinus thrombosis-emerging new causes and treatments. J Thromb Haemost. (2024) 22:3346–54. doi: 10.1016/j.jtha.2024.08.012, 39260741

[12] Ferro JM Canhão P Stam J Bousser MG Barinagarrementeria F. Prognosis of cerebral vein and dural sinus thrombosis: results of the international study on cerebral vein and Dural sinus thrombosis (ISCVT). Stroke. (2004) 35:664–70. doi: 10.1161/01.STR.0000117571.76197.26 14976332

[13] Klein P Shu L Nguyen TN Siegler JE Omran SS Simpkins AN . Outcome prediction in cerebral venous thrombosis: the IN-REvASC score. J Stroke. (2022) 24:404–16. doi: 10.5853/jos.2022.01606, 36221944 PMC9561213

[14] Eskey CJ Meyers PM Nguyen TN Ansari SA Jayaraman M McDougall CG . Indications for the performance of intracranial endovascular Neurointerventional procedures: a scientific statement from the American Heart Association. Circulation. (2018) 137:e661–89. doi: 10.1161/CIR.0000000000000567, 29674324

[15] Aamodt AH Skattør TH. Cerebral venous thrombosis. Semin Thromb Hemost. (2022) 48:309–17. doi: 10.1055/s-0042-1742738, 35170006

[16] Ferro JM Coutinho JM Dentali F Kobayashi A Alasheev A Canhão P . Safety and efficacy of dabigatran Etexilate vs dose-adjusted warfarin in patients with cerebral venous thrombosis: a randomized clinical trial. JAMA Neurol. (2019) 76:1457–65. doi: 10.1001/jamaneurol.2019.2764, 31479105 PMC6724157

[17] Field TS Lindsay MP Wein T Debicki DB Gorman J Heran MKS . Canadian stroke best practice recommendations, 7(th) edition: cerebral venous thrombosis. Can J Neurol Sci. (2024) 52:370–17. doi: 10.1017/cjn.2024.350, 39639491

[18] Coutinho JM Zuurbier SM Bousser M-G Ji X Canhão P Roos YB . Effect of endovascular treatment with medical management vs standard care on severe cerebral venous thrombosis: the TO-ACT randomized clinical trial. JAMA Neurol. (2020) 77:966–73. doi: 10.1001/jamaneurol.2020.1022, 32421159 PMC7235912

[19] Connor P Sánchez van Kammen M Lensing AWA Chalmers E Kállay K Hege K . Safety and efficacy of rivaroxaban in pediatric cerebral venous thrombosis (EINSTEIN-Jr CVT). Blood Adv. (2020) 4:6250–8. doi: 10.1182/bloodadvances.2020003244, 33351120 PMC7756994

[20] Branson SV McClintic E Yeatts RP. Septic cavernous sinus thrombosis associated with orbital cellulitis: a report of 6 cases and review of literature. Ophthalmic Plast Reconstr Surg. (2019) 35:272–80. doi: 10.1097/IOP.0000000000001231, 30320718

[21] Smith DM Vossough A Vorona GA Beslow LA Ichord RN Licht DJ. Pediatric cavernous sinus thrombosis: a case series and review of the literature. Neurology. (2015) 85:763–9. doi: 10.1212/WNL.0000000000001886, 26231260 PMC4553026

[22] Khatri IA Wasay M. Septic cerebral venous sinus thrombosis. J Neurol Sci. (2016) 362:221–7. doi: 10.1016/j.jns.2016.01.035 26944152

[23] Wang YH Chen PY Ting PJ Huang FL. A review of eight cases of cavernous sinus thrombosis secondary to sphenoid sinusitis, including a12-year-old girl at the present department. Infect Dis (Lond). (2017) 49:641–6. doi: 10.1080/23744235.2017.1331465, 28535728

[24] Battistin U Hallak YO Hallak F Hallak O AlKhani R. Subacute cavernous sinus thrombosis following a dental procedure: case report and review of the literature. Clin Neurol Neurosurg. (2020) 197:106135. doi: 10.1016/j.clineuro.2020.106135, 32836064

[25] Hsu CW Tsai WC Lien CY Lee JJ Chang WN. The clinical characteristics, implicated pathogens and therapeutic outcomes of culture-proven septic cavernous sinus thrombosis. J Clin Neurosci. (2019) 68:111–6. doi: 10.1016/j.jocn.2019.07.022, 31331748

[26] Raja K Parida PK Alexander A Surianarayanan G. Otogenic lateral sinus thrombosis: a review of fifteen patients and changing trends in the management. Int Arch Otorhinolaryngol. (2018) 22:208–13. doi: 10.1055/s-0037-1604198, 29983756 PMC6033598

[27] de Oliveira PN Testa JRG Inoue DP Cruz OLM. Presentation, treatment, and clinical course of otogenic lateral sinus thrombosis. Acta Otolaryngol. (2009) 129:729–34. doi: 10.1080/00016480802399721, 18781447

[28] Sitton MS Chun R. Pediatric otogenic lateral sinus thrombosis: role of anticoagulation and surgery. Int J Pediatr Otorhinolaryngol. (2012) 76:428–32. doi: 10.1016/j.ijporl.2011.12.025, 22277267

[29] Kuczkowski J Narozny W Mikaszewski B. Lateral sinus thrombosis in chronic otitis media. Otol Neurotol. (2007) 28:992–3. doi: 10.1097/01.MAO.0000271713.91100.d2, 17909438

[30] Field TS Lindsay MP Wein T Debicki DB Gorman J Heran MKS . Canadian stroke best practice recommendations, 7thEdition: cerebral venous thrombosis, 2024. Can J Neurol Sci. (2025) 52:353–69. doi: 10.1017/cjn.2024.269, 38826076

[31] Yuan L Yuan J Sun Y Wang Y. The anticoagulant therapy for Otogenic sigmoid sinus thrombophlebitis: a case report and literature review. Ear Nose Throat J. (2022) 101:NP379–82. doi: 10.1177/0145561320976405, 33236644

[32] DiSciullo A Gerges D Arnell TL Herrington H. Bilateral lateral sinus thrombosis secondary to acute mastoiditis. Otolaryngol Case Rep. (2020) 17:100219. doi: 10.1016/j.xocr.2020.100219

[33] Umurungi J Ferrando F Cilloni D Sivera P. Cerebral vein thrombosis and direct Oral anticoagulants: a review. J Clin Med. (2024) 13:4730. doi: 10.3390/jcm13164730, 39200872 PMC11355492

[34] Miller TR Gandhi D. Intracranial Dural Arteriovenous Fistulae. Stroke. (2015) 46:2017–25. doi: 10.1161/STROKEAHA.115.008228, 25999384

[35] Abdalkader M Nguyen TN Diana F Yaghi S Shu L Klein P . Intracranial Dural Arteriovenous Fistulas. Semin Neurol. (2023) 43:388–96. doi: 10.1055/s-0043-1771453, 37562448

[36] Brinjikji W Cloft HJ Lanzino G. Clinical presentation and imaging findings of patients with Dural arteriovenous fistulas with an angiographic Pseudophlebitic pattern. Am J Neuroradiol. (2020) 41:2285–91. doi: 10.3174/ajnr.A6811, 33093135 PMC7963232

[37] Su X Sun L Gao Y Song Z Chen Y Zhang H . Clinical outcomes of intracranial dural arteriovenous fistulas with the pseudophlebitic pattern. Journal of NeuroInterventional. Surgery. (2025). doi: 10.1136/jnis-2025-024390, 41136213

[38] Kurisu K Osanai T Morishima Y Ito M Uchino H Sugiyama T . Systemic immune-inflammation index in dural arteriovenous fistula: a feasible biomarker reflecting its clinical characteristics. Acta Neurochir. (2024) 166:180. doi: 10.1007/s00701-024-06075-1, 38627314

[39] Tritanon O Khunvutthidee S Kobkitsuksakul C Jindahra P Panyaping T. Differentiation between aggressive and benign intracranial non-cavernous dural arteriovenous fistulas using cortical venous reflux on susceptibility weighted images. Eur J Radiol. (2023) 162:110800. doi: 10.1016/j.ejrad.2023.110800, 36990052

[40] Zhang C Su X Song Z Liu H Zhang H Zhang P . Prognostic value of novel inflammatory indices in dural arteriovenous fistula patients undergoing endovascular treatment. J NeuroInt Surg. (2025):jnis-2025-023792. doi: 10.1136/jnis-2025-023792, 40830051

[41] Chen Zhou ZH Hilario A Salvador Álvarez E Cárdenas Del Carre AM Romero Coronado J Lechuga Vázquez C . The "Hypointense focal brain" on susceptibility-weighted imaging as a sign of venous congestion in cranial dural arteriovenous fistulas. Neuroradiol J. (2025) 38:64–71. doi: 10.1177/19714009241269522, 39075737 PMC11571536

[42] Sivan Sulaja J Kannath SK Ganesh KSV Thomas B. Evaluation of multiple deep neural networks for detection of intracranial dural arteriovenous fistula on susceptibility weighted angiography imaging. Neuroradiol J. (2025) 38:72–8. doi: 10.1177/19714009241269491, 39089849 PMC11571296

[43] Maciejewski K Pinkiewicz M Mruk B Knap D Zaczyński A Walecki J . A practical approach to intracranial Dural arteriovenous fistulas: pathogenesis, classification and management. J Clin Med. (2025) 14:6895. doi: 10.3390/jcm14196895, 41095985 PMC12524896

[44] Guniganti R Giordan E Chen CJ Abecassis IJ Levitt MR Durnford A . Consortium for Dural arteriovenous fistula outcomes research (CONDOR): rationale, design, and initial characterization of patient cohort. J Neurosurg. (2022) 136:951–61. doi: 10.3171/2021.1.JNS202790, 34507282

[45] Wall M Kupersmith MJ Kieburtz KD Corbett JJ Feldon SE Friedman DI . The idiopathic intracranial hypertension treatment trial: clinical profile at baseline. JAMA Neurol. (2014) 71:693–701. doi: 10.1001/jamaneurol.2014.133, 24756302 PMC4351808

[46] Thurtell MJ. Idiopathic intracranial hypertension. Continuum (Minneap Minn). (2019) 25:1289–309. doi: 10.1212/CON.0000000000000770 31584538

[47] Schartz D Finkelstein A Bender M Kessler A Zhong J. Association of Extent of transverse sinus stenosis with cerebral Glymphatic clearance in patients with idiopathic intracranial hypertension. Neurology. (2024) 103:e209529. doi: 10.1212/WNL.0000000000209529, 38833652

[48] Zhao K Gu W Liu C Kong D Zheng C Chen W . Advances in the understanding of the complex role of venous sinus stenosis in idiopathic intracranial hypertension. J Magn Reson Imaging. (2022) 56:645–54. doi: 10.1002/jmri.28177, 35357056 PMC9541264

[49] Fargen KM Liu K Garner RM Greeneway GP Wolfe SQ Crowley RW. Recommendations for the selection and treatment of patients with idiopathic intracranial hypertension for venous sinus stenting. J Neurointerv Surg. (2018) 10:1203–8. doi: 10.1136/neurintsurg-2018-014042, 30030306

[50] Fargen KM Amans MR Hui FK. The cerebral venous disorders severity scale. J NeuroInt Surg. (2025) 17:115–6. doi: 10.1136/jnis-2024-022976, 39824604

[51] Wu AQ Gitlevich R Palepu C Hoglund ZT Sollenberger CH Kahlon AS . Mapping real-time hemodynamic changes in venous sinus stenting using intraoperative transcranial Doppler ultrasonography and quantitative angiography. Neurosurg Focus. (2026) 60:E10. doi: 10.3171/2025.10.FOCUS25838, 41569736

[52] Ahmad SR Moss HE. Update on the diagnosis and treatment of idiopathic intracranial hypertension. Semin Neurol. (2019) 39:682–91. doi: 10.1055/s-0039-1698744, 31847039 PMC7713505

[53] Shields LBE Shields CB Yao TL Plato BM Zhang YP Dashti SR. Endovascular treatment for venous sinus stenosis in idiopathic intracranial hypertension: An observational study of clinical indications, surgical technique, and Long-term outcomes. World Neurosurg. (2019) 121:e165–71. doi: 10.1016/j.wneu.2018.09.070, 30248468

[54] Friedman DI Liu GT Digre KB. Revised diagnostic criteria for the pseudotumor cerebri syndrome in adults and children. Neurology. (2013) 81:1159–65. doi: 10.1212/WNL.0b013e3182a55f17, 23966248

[55] Mollan SP Davies B Silver NC Shaw S Mallucci CL Wakerley BR . Idiopathic intracranial hypertension: consensus guidelines on management. J Neurol Neurosurg Psychiatry. (2018) 89:1088–100. doi: 10.1136/jnnp-2017-317440, 29903905 PMC6166610

[56] Townsend RK Fargen KM. Intracranial venous hypertension and venous sinus stenting in the modern Management of Idiopathic Intracranial Hypertension. Life. (2021) 11:508 doi: 10.3390/life11060508, 34073077 PMC8227267

[57] Guo X-b Wei S Guan S. Intracranial venous pressures manometry for patients with idiopathic intracranial hypertension: under awake setting or general anesthesia. Front Neurol. (2019) 10:751. doi: 10.3389/fneur.2019.00751, 31354615 PMC6640650

[58] Zhang Y Ma C Li C Li X Liu R Liu M . Prediction of the trans-stenotic pressure gradient with arteriography-derived hemodynamic features in patients with idiopathic intracranial hypertension. J Cereb Blood Flow Metab. (2022) 42:1524–33. doi: 10.1177/0271678X221086408, 35255760 PMC9274861

[59] Committee TNIIHSGW. Effect of acetazolamide on visual function in patients with idiopathic intracranial hypertension and mild visual loss: the idiopathic intracranial hypertension treatment trial. JAMA. (2014) 311:1641–51. doi: 10.1001/jama.2014.3312, 24756514 PMC4362615

[60] Azzam AY Mortezaei A Morsy MM Essibayi MA Ghozy S Elamin O . Venous sinus stenting for idiopathic intracranial hypertension: An updated Meta-analysis. J Neurol Sci. (2024) 459:122948. doi: 10.1016/j.jns.2024.122948, 38457956

[61] Patsalides A Fargen KM Davies JM Boddu SR Dinkin M Priest R . The River study: the first prospective multicenter trial of a novel venous sinus stent for the treatment of idiopathic intracranial hypertension. J NeuroInt Surg. (2026) 18:11–9. doi: 10.1136/jnis-2024-022540 39978822

[62] Midtlien JP Kittel C Klever LA Kiritsis NR Aldridge JB Fargen KM. Redefining treatment expectations: exploring mid- and long-term outcomes of venous sinus stenting in idiopathic intracranial hypertension. J Neurointerv Surg. (2025) 17:215–21. doi: 10.1136/jnis-2023-021336, 38453459

[63] Handzic A Xie JS Hendriks E Mosimann P Nicholson P Micieli J . Visual and pharmacotherapy outcomes after transverse sinus stenting for idiopathic intracranial hypertension. [Epub ahed of print] (2023) 10.1097/WNO.0000000000002156 38627888

[64] Dinkin MJ Patsalides A. Idiopathic intracranial venous hypertension: toward a better understanding of venous stenosis and the role of stenting in idiopathic intracranial hypertension. J Neuroophthalmol. (2023) 43:451–63. doi: 10.1097/WNO.0000000000001898, 37410913

[65] Abdalkader M Nguyen TN Norbash AM Raz E Shapiro M Lenck S . State of the art: venous causes of pulsatile tinnitus and diagnostic considerations guiding endovascular therapy. Radiology. (2021) 300:2–16. doi: 10.1148/radiol.2021202584, 34032509

[66] Essibayi MA Oushy SH Lanzino G Brinjikji W. Venous causes of pulsatile tinnitus: clinical presentation, clinical and radiographic evaluation, pathogenesis, and endovascular treatments: a literature review. Neurosurgery. (2021) 89:760–8. doi: 10.1093/neuros/nyab299, 34392338

[67] Pandey A Schreiber C Garton ALA Jung B Goldberg JL Kocharian G . Challenges in the use of venous sinus stenting in the treatment of idiopathic intracranial hypertension and pulsatile tinnitus. World Neurosurg. (2024) 184:372–86. doi: 10.1016/j.wneu.2023.12.164, 38590071

[68] Narsinh KH Hui F Saloner D Tu-Chan A Sharon J Rauschecker AM . Diagnostic approach to pulsatile tinnitus: a narrative review. JAMA Otolaryngol Head Neck Surg. (2022) 148:476–83. doi: 10.1001/jamaoto.2021.4470, 35201283

[69] Cavarocchi C Wong K Cao AC Hwa TP Quimby AE Eliades SJ . Toward a diagnostic imaging algorithm for undifferentiated pulsatile tinnitus. Otol Neurotol. (2024) 45:895–900. doi: 10.1097/MAO.0000000000004254, 39052898

[70] Jain V Policeni B Juliano AF Adunka O Agarwal M Dubey P . ACR appropriateness criteria® tinnitus: 2023 update. J Am Coll Radiol. (2023) 20:S574–91. doi: 10.1016/j.jacr.2023.08.017, 38040471

[71] Verbist B Connor S Farina D. ESR essentials: diagnostic strategies in tinnitus-practice recommendations by the European Society of Head and Neck Radiology. Eur Radiol. (2025) 35:1303–12. doi: 10.1007/s00330-024-11316-z, 39747584

[72] Wang AC Nelson AN Pino C Svider PF Hong RS Chan E. Management of Sigmoid Sinus Associated Pulsatile Tinnitus: a systematic review of the literature. Otol Neurotol. (2017) 38:1390–6. doi: 10.1097/MAO.0000000000001612, 29135862

[73] Boddu S Dinkin M Suurna M Hannsgen K Bui X Patsalides A. Resolution of pulsatile tinnitus after venous sinus stenting in patients with idiopathic intracranial hypertension. PLoS One. (2016) 11:e0164466. doi: 10.1371/journal.pone.0164466, 27768690 PMC5074492

[74] Farid M Alawamry A Zaitoun MMA Bessar AA Darwish EAF. Relentless pulsatile tinnitus secondary to dural sinovenous stenosis: is endovascular sinus stenting the answer? Clin Radiol. (2021) 76:526–31. doi: 10.1016/j.crad.2021.02.022, 33757666

[75] Schartz D Finkelstein A Akkipeddi SMK Williams Z Vates E Bender MT. Outcomes of pulsatile tinnitus after cerebral venous sinus stenting: systematic review and pooled analysis of 616 patients. World Neurosurg. (2024) 190:e992–9. doi: 10.1016/j.wneu.2024.08.048, 39142383

[76] An Y-H Han S Lee M Rhee J Kwon OK Hwang G . Dural arteriovenous fistula masquerading as pulsatile tinnitus: radiologic assessment and clinical implications. Sci Rep. (2016) 6:36601. doi: 10.1038/srep36601 27812001 PMC5095646

[77] Cummins DD Caton MT Hemphill K Lamboy A Tu-Chan A Meisel K . Cerebrovascular pulsatile tinnitus: causes, treatments, and outcomes in 164 patients with neuroangiographic correlation. J Neurointerv Surg. (2023) 15:1014–20. doi: 10.1136/jnis-2022-019259, 36190940

[78] San Millán Ruíz D Delavelle J Yilmaz H Gailloud P Piovan E Bertramello A . Parenchymal abnormalities associated with developmental venous anomalies. Neuroradiology. (2007) 49:987–95. doi: 10.1007/s00234-007-0279-0 17703296

[79] Akers A Al-Shahi Salman R Issam AA Dahlem K Flemming K Hart B . Synopsis of guidelines for the clinical Management of Cerebral Cavernous Malformations: consensus recommendations based on systematic literature review by the Angioma Alliance scientific advisory board clinical experts panel. Neurosurgery. (2017) 80:665–80. doi: 10.1093/neuros/nyx091 28387823 PMC5808153

[80] Bateman GA. The pathophysiology of idiopathic normal pressure hydrocephalus: cerebral ischemia or altered venous hemodynamics? AJNR Am J Neuroradiol. (2008) 29:198–203. doi: 10.3174/ajnr.A0739, 17925373 PMC8119093

[81] Farb RI Vanek I Scott JN Mikulis DJ Willinsky RA Tomlinson G . Idiopathic intracranial hypertension: the prevalence and morphology of sinovenous stenosis. Neurology. (2003) 60:1418–24. doi: 10.1212/01.WNL.0000066683.34093.E2 12743224

[82] Dinkin M Oliveira C. Men are from Mars, idiopathic intracranial hypertension is from venous: the role of venous sinus stenosis and stenting in idiopathic intracranial hypertension. Semin Neurol. (2019) 39:692–703. doi: 10.1055/s-0039-3399506 31847040

[83] Satti SR Leishangthem L Chaudry MI. Meta-analysis of CSF diversion procedures and Dural venous sinus stenting in the setting of medically refractory idiopathic intracranial hypertension. AJNR Am J Neuroradiol. (2015) 36:1899–904. doi: 10.3174/ajnr.A4377 26251432 PMC7965019

[84] Yaghi S Saldanha IJ Misquith C Zaidat B Shah A Joudi K . Direct Oral anticoagulants versus vitamin K antagonists in cerebral venous thrombosis: a systematic review and Meta-analysis. Stroke. (2022) 53:3014–24. doi: 10.1161/STROKEAHA.122.039579 35938419

[85] Ferro JM Bousser MG Canhão P Coutinho JM Crassard I Dentali F . European stroke organization guideline for the diagnosis and treatment of cerebral venous thrombosis - endorsed by the European academy of neurology. Eur J Neurol. (2017) 24:1203–13. doi: 10.1111/ene.13381 28833980

[86] Inam ME Martinez-Gutierrez JC Kole MJ Sanchez F Lekka E Truong VTT . Venous sinus stenting for low pressure gradient Stenoses in idiopathic intracranial hypertension. Neurosurgery. (2022) 91:734–40. doi: 10.1227/neu.0000000000002095, 35960743 PMC10553007

